# Fabellar prevalence, degeneration and association with knee osteoarthritis in the Chinese population

**DOI:** 10.1038/s41598-019-49174-1

**Published:** 2019-09-10

**Authors:** Weikun Hou, Lin Xu, Jingbo Wang, Bo Wang, Lin Liu, Ke Xu, Yuanzhen Cai, Hao Guo, Peng Xu

**Affiliations:** 1 Osteonecrosis and Joint Reconstruction Ward, Department of Joint Surgery, HongHui Hospital, Xi’an Jiaotong University Health Science Center, Xi’an, Shaanxi 710054 China; 2 Department of Orthopaedics, Hanzhong 3201 Hospital, Hanzhong, Shaanxi 723000 China; 3 Department of Radiology, HongHui Hospital, Xi’an Jiaotong University Health Science Center, Xi’an, Shaanxi 710054 China; 4 Center for Translational Medicine, The First Affiliated Hospital of Xi’an Jiaotong University, Xi’an, Shaanxi 710061 China

**Keywords:** Osteoarthritis, Radiography

## Abstract

The fabella is a sesamoid bone of the knee that can degenerate in some patients with osteoarthritis. The purpose of this study was to examine the prevalence and degeneration grades of fabellae in the Chinese population and to analyse their relationships with subject ages and knee osteoarthritis grades. The anteroposterior and lateral knee roentgenograms of 1150 subjects were recruited from the institutional database. The Kellgren-Lawrence scoring system was used to evaluate knee osteoarthritis. The degeneration grades of fabellae were scored in lateral roentgenograms by screening their shapes, sizes, subchondral sclerosis and osteophyte formation. The prevalence and degeneration of fabellae among ages, genders and knee sides were analysed by the Pearson Chi-Square test, and their relationships with knee osteoarthritis were analysed by the Spearman nonparametric correlation test. The overall prevalence of fabellae was 48.6% in 1359 knees. There was no significant difference in fabellar prevalence between the two sides (*χ*² = 0.025, *P* = 0.87437) and genders (*χ*² = 3.647, *P* = 0.05617), while the prevalence increased with the increasing ages of the subjects (*χ*² = 213.868, *P* < *0.001*). The fabellar degeneration grades were correlated with age (*r* = 0.5288, *P* < 0.001) and knee osteoarthritis scores (*r* = *0.6892, P* < *0.001*). These results suggested that the fabellar prevalence and degeneration grades were correlated with age and knee osteoarthritis scores.

## Introduction

The fabella is a small fibrocartilaginous body or sesamoid bone embedded in the tendon of the lateral head of the gastrocnemius muscle and articulated with the posterior surface of the lateral condyle of the femur. The fabella can prevent friction-induced damage to the tendon, increase the efficiency of the gastrocnemius muscle and cooperate with the fabellofibular ligament to stabilize the posterolateral part of the knee^1–3^.

The prevalence of fabellae varies among global regions, ethnicities and observational methods from 3.1% to 86.9%^4,5^. Previous studies have shown that Eastern people, older people and knee osteoarthritis (KOA) patients more frequently to presented with fabellae^2,3,5–7^.

The fabella is usually a benign structure; however, in rare cases, it involves disorders, such as chondromalacia, osteoarthritis, dislocation and fracture, resulting in fabella syndrome or popliteal artery entrapment syndrome, which influences the common fibular nerve or popliteal artery^8–13^. These fabellar disorders and fabellar impingement with prostheses can lead to knee pain after total knee arthroplasty (TKA), which should attract the concerns of orthopaedists because of the increasing number and anticipation of TKA patients^14–16^.

However, there are few fabella-related studies, most of which are case reports or small sample size studies. There is no reported fabella degeneration scoring system, and it is unclear whether fabellar prevalence and degeneration is correlated with knee osteoarthritis. In this study, the prevalence of fabellae among genders, ages and knee sides in the knee roentgenograms of the Chinese population was examined, and the relationships between fabellar prevalence or degeneration grades and ages or knee osteoarthritis grades were analysed.

## Methods

### Subjects

This retrospective observational study was approved by the Biomedical Research Ethics Committee of HongHui Hospital, Xi’an Jiaotong University, and written informed consent was waived. All methods and procedures were performed in accordance with the relevant guidelines and regulations. Archival records from July 2016 to August 2017 in our institutional inpatients and clinic database were used. A total of 1150 patients (462 male and 688 female subjects, aged from 21 months to 86 years, 1359 knee roentgenograms) were recruited. All subjects had both anteroposterior and lateral knee radiographs. The exclusion conditions before analysis were as follows: difficult to discriminate the fabella and posterior osteophytes in patients with advanced osteoarthritis, the posterior area of the knee overlapped with the femoral condyle or femoral prosthesis or other internal fixations because of the rotation of lateral radiographs.

All radiographs were reviewed by two orthopaedists independently, and the final decision was reached with consensus after discussion in uncertain cases. Information, including the subject age, gender, and side of the knee were retrieved from institutional database records.

### Radiographic scorings of knee osteoarthritis and fabella degeneration

**Figure 1:**
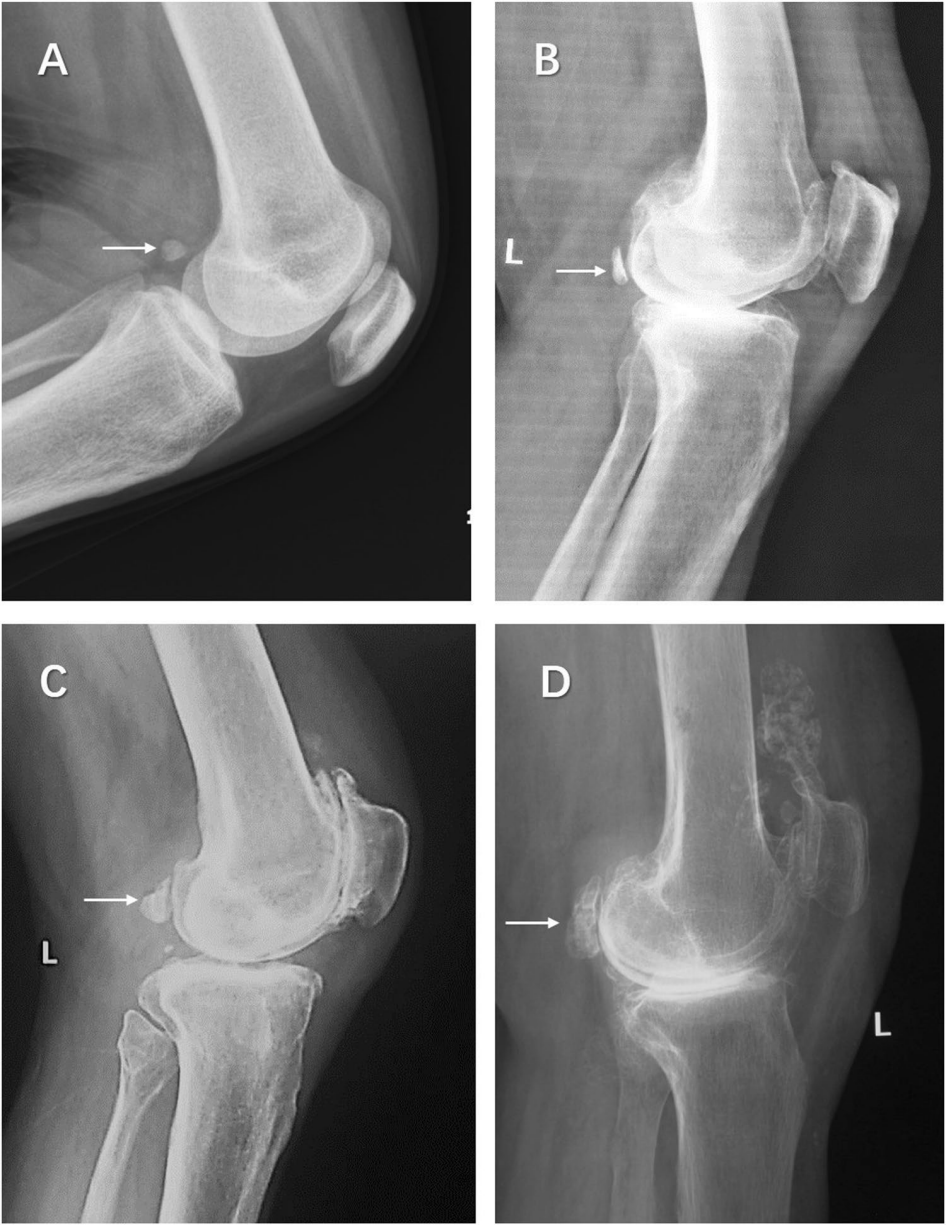
Different degenerated grades of fabellae are shown in lateral knee radiographies. **(A**) Grade 0, normal fabella, which is oval shaped, and anterior smooth surface is articulated with posterolateral condyle of femur; (**B**) Grade 1, sclerosis fabella, with anterior surface subchondral sclerosis of the fabella; (**C**) Grade 2, severe sclerosis of the fabella with osteophyte formation; (**D**) Grade 3, large fabella with marked osteophyte formation. Arrows point to the fabellae.

The Kellgren-Lawrence radiological grading scheme was used to assess knee osteoarthritis^17^. The degeneration grades of fabellae were scored by screening their shapes, sizes, anterior surface subchondral sclerosis and osteophyte formation as follows: Grade 0, normal fabella with triangular or oval shape, possibly having a smooth anterior surface articulating with a posterior femoral condyle; Grade 1, sclerotic fabella with anterior surface subchondral sclerosis; Grade 2, severe sclerotic fabella with osteophyte formation on the fabellar margins; and Grade 3, huge fabella (maximum length over 2 cm) with large osteophyte formation (Fig. 1).

### Statistical methods

Continuous variables were stated as the mean and standard deviation, and categorical variables were stated as percentages and frequency distributions. The differences in fabellar prevalence among genders, knee sides and ages were analysed by Pearson Chi-Square test. The Spearman nonparametric correlation test was used for correlative analysis. A *P*-value < 0.05 was considered statistically significant.

## Results

A total of 1150 subjects were included, with 462 males and 688 females aged from 21 months to 86 years (43.9 ± 18.8, mean ± SD, years), 908 subjects had osteoarthritis and 242 did not. There were 1359 knees examined, including 209 subjects with bilateral knee radiographs and 941 subjects with unilateral knee radiographs (449 left knees and 492 right knees).

The overall fabellar prevalence was 48.6% in 1359 knees. There was no significant gender difference in fabellar prevalence, with 45.3% in males and 50.6% in females (*χ*² = 3.647, *P* = 0.05617). There was no significant difference in fabellar prevalence between the two sides, with 48.8% in left knees and 48.4% in right knees (*χ*² = 0.025, *P* = 0.87437, Table 1). The fabellar prevalence was 55.5% in knees with osteoarthritis, which was higher than the 21.4% prevalence in knees without osteoarthritis (*χ*² = 102.490, *P* < 0.0001, Table 1).

**Table 1:** The fabellar prevalence among genders, knee sides and osteoarthritis.

^a^Number (%)		Absent	Present	*χ*²	*P*
Gender	Male	283 (54.7%)	234 (45.3%)	3.647	0.05617
	female	416 (49.4%)	426 (50.6%)		
Leg	Left	337 (51.2%)	321 (48.8%)	0.025	0.87437
	Right	362 (51.6%)	339 (48.4%)		
Condition of the knee	Normal	217 (78.6%)	59 (21.4%)	102.490	<0.0001^*^
	OA	482 (44.5%)	601 (55.5%)		
Total		699 (51.4%)	660 (48.6%)		

^a^Denotes percentage of knees;

*Denotes significance at *P* < 0.05.

The subjects were subdivided into five different age groups. The prevalence of fabellae was significantly different among these age groups, with 8.2% in the ≤20 years age group, 36.4% in the 21–35 years age group, 47.9% in the 36–50 years age group, 58.2% in the 51–65 years age group and 79.3% in the ≥66 years age group (*χ*² = 213.868, *P* < 0.001). The prevalence of fabellae was correlated with age, which increased from young to old age groups (*r* = 0.3847, *P* < 0.001, Table 2).

**Table 2:** The fabellar prevalence among different age groups.

	Ages	Absent		Present	
		^a^Number	%	^a^Number	%
Age groups	≤20	146	91.8%	13	8.2%
	21–35	159	63.6%	91	36.40
	36–50	220	52.1%	202	47.9%
	51–65	128	41.8%	178	58.2%
	≥66	46	20.7%	176	79.3%
Chi-square test	*χ²*	213.868			
	*P*	<0.001^*^			
Spearman’s nonparametric correlation test	*r*	0.3847			
	*P’*	<0.001^*^			

^a^Denotes the number of knees;

^*^Denotes significance at *P* < 0.05.

The degeneration grades of fabellae in different age groups were evaluated. The degeneration grades were significantly different among age groups (*χ*² = 208.954, *P* < 0.001); the number of degenerated fabella was 0 out of 13 fabellae in the ≤20 years age group, 18 out of 91 fabellae in the 21–35 years age group, 86 out of 202 fabellae in the 36–50 years age group, 113 out of 178 fabellae in the 51–65 years age group, and 160 out of 186 fabellae in the ≥66 years age group. The subject age was correlated with the degeneration grades of fabellae, and older subjects presented more severe degenerated fabellae (*r* = 0.5288, *P* < 0.001, Table 3).

**Table 3:** The degenerated grades of fabellae among different age groups.

	^a^Number	FDG 0	FDG 1	FDG 2	FDG 3
Age groups	≤20	13	0	0	0
	21–35	73	16	2	0
	36–50	116	60	23	3
	51–65	65	46	52	15
	≥66	26	43	81	36
Chi-square test	*χ²*	208.954			
	*P*	<0.001^*^			
Spearman nonparametric correlation test	*r*	0.5288			
	*P*	<0.001^*^			

FDG, fabella degeneration grade; ^a^Denotes the number of knees;

^*^Denotes significance at *P* < 0.05.

**Figure 2:**
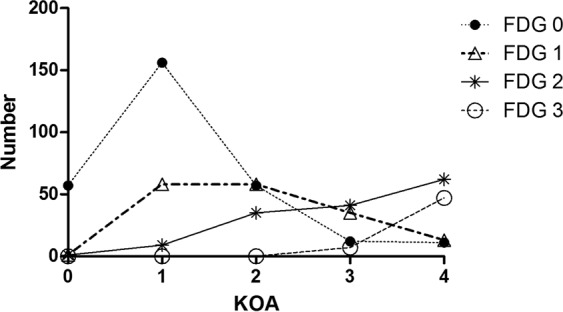
The relationship between fabella degeneration grades and knee osteoarthritis Kellgren-Lawrence grades. Spearman’s nonparametric correlation test was used (*r* = 0.6892, *P* < 0.001), and a *P*-value < 0.05 was considered statistically significant. FDG, fabella degeneration grade; KOA, knee osteoarthritis Kellgren-Lawrence grade.

The degeneration grades of fabellae were significantly different among knee osteoarthritis conditions (*χ²* = 426.112, *P* < 0.001). In knees without osteoarthritis (KOA 0 group), there were 57 normal fabellae (degeneration grade 0), 1 fabella with degeneration grade 1, 1 fabella with degeneration grade 2 and no fabella with degeneration grade 3; in knees with Kellgren-Lawrence grade 1 osteoarthritis (KOA 1 group), the numbers of fabellae were 156, 58, 9 and 0 with degeneration grades 0, 1, 2 and 3, respectively; in KOA 2 group, the numbers of fabellae were 57, 58, 35 and 0 with gradually increased degeneration grades; in the KOA 3 group, the numbers of different degenerated fabellae were 12, 35, 41 and 7, while in the KOA 4 group, these numbers were 11, 13, 62 and 47, respectively (Table 4). The degeneration grades of fabellae were positively correlated with the osteoarthritis grades in the same knee (*r* = *0.6892*, *p* < 0.001, Fig. 2).

**Table 4:** Degenerated grades of fabellae correlated with knee osteoarthritis grades.

	^a^Number	FDG 0	FDG 1	FDG 2	FDG 3
Groups	KOA 0	57	1	1	0
	KOA 1	156	58	9	0
	KOA 2	57	58	35	0
	KOA 3	12	35	41	7
	KOA 4	11	13	62	47
Chi-square test	*χ²*	426.112			
	*P*	<0.001^*^			

FDG, fabella degeneration grade;

KOA, knee osteoarthritis Kellgren-Lawrence grade; ^a^Denotes the number of knees;

^*^Denotes significance at *P* < 0.05.

## Discussion

The fabella, similar to other sesamoid bones, appears as a cartilaginous nodule in the foetus under substantial genetic control and consequently ossifies under compressive load on the lateral gastrocnemius tendon by endochondral ossifications^18–20^.

We found that the prevalence of fabellae in the Chinese population screened by radiology was 48.6%, which is consistent with the scale of published reports on the Asian population. The reported fabellar prevalence varied among different observational methods, ethnic populations, ages and degeneration of the knee^21–25^.

The observational methods included radiological (roentgenogram or CT), MRI and cadaver studies. The sensitivity of radiological methods were similar in studies on bony fabellae while lower in studies on cartilage fabellae in comparison to those of MRI or anatomic methods^5,26^. CT and MRI are more cost-consuming, while excising fabella or cadaver are not suitable for most patients, which restricts the clinical usage of these methods. Roentgenogram is the most popular method for musculoskeletal disorder diagnosis and an effective method for grading.

The prevalence of fabellae was significantly different among ethnic populations, from 3.1% to 31.3% in Caucasian individuals and from 30.6% to 92% in Asian individuals (fabella/knee, Table 5). The assumed cause was lifestyle, while there were no related genetic studies. For example, Asians prefer kneeling, squatting and tailor sitting, which can give persistent pressure of the fabella against the posterior aspect of the lateral femoral condyle and promote fabella development and ossification^27^.

**Table 5:** Prevalence of fabellae among different reports.

Author	Year	Ethnic group	Method	Ages	Fabella/knee	Incidence
Silva^4^	2010	Caucasian (Brazil)	Cadaver	38–78 y	2/64	3.1
Kaplan^21^	1961	Caucasian (America)	Cadaver	NA	10/135	8.7
Raheem^22^	2007	Caucasian (Ireland)	Cadaver	84 ± 8.1 y^a^	2/22	9.1
Phukubye^29^	2011	Caucasian and African	Cadaver	40–95 y	18/102	17.6
Yu^23^	1996	Caucasian (America)	MRI	12–72 y	19/100	19.0
Terry^24^	1996	Caucasian (Georgia)	Radiography	NA	5/25	20
Egerci^31^	2016	Caucasian (Turkey)	Radiography	18–90 y	228/1000	22.8
Pritchett^7^	1984	Caucasian (America)	Radiography	31–87 y^*^	252/972	25.9
Hauser^2^	2015	European (Switzerland)	CT	20–104 y	105/400	26.3
Ehara^26^	2014	Asian (Japan)	MRI	4–89 y	200/653	30.6
Sarin^20^	1999	Caucasian (America)	Radiography	19–84 y	45/112	31.3
Chew^27^	2014	Asian (Singapore)	Radiography and MRI	14–55 y	25/80	31.3
Piyawinijwong^25^	2012	Asian (Thailand)	Cadaver	30–97 y	144/372	38.7
Present study	2017	Asian (China)	Radiograph	21 m^†^–86 y	660/1359	48.6
Tabira^30^	2013	Asian (Japan)	Cadaver	74.5 ± 12.3 y^a^	70/102	68.6
Minowa^6^	2004	Asian (Japan)	Cadaver	54–98 y	182/212	85.8
Zeng^5^	2012	Asian (China)	Cadaver and Radiography	64–83 y	53/61	86.9
Kawashima^3^	2007	Asian (Japan)	Cadaver	66–100 y	69/75	92.0

y^*^ Denotes years;

m^†^ Denotes months;

^a^Denotes mean ± SD.

The relationship between age and fabellar prevalence remains controversial. In this study, a large sample size of subjects was recruited, and a high prevalence rate of fabellae was observed; the fabellar prevalence was positively correlated with age. Similar reports by Iida H *et al*. showed that the fabellar prevalence increased with age^28^. However, Phukubye P *et al*. found that the prevalence of fabellae was 11.1% in the 40–49 year age group, 22.9% in the 70–89 year age group and 42.9% in the >90 year age group. Although there was an increasing trend, these authors did not find a significant correlation between age and fabellar prevalence. The cause was assumed to be small sample size, with only 18 fabellae found in their research^29^. Tabira Y *et al*. reported that age was not associated with the frequency of fabellae, while their subjects involved only 102 knees^30^. Egerci OF *et al*. also recently reported no relationship between age and fabellar prevalence in the Turkish population by radiological method^31^. As cartilaginous fabella cannot be distinguished by radiological methods, further studies by MRI or anatomic methods would be beneficial to explain these controversial results.

Osteoarthritis was also considered an influencing factor for fabellar prevalence. Pritchett JW *et al*. first studied the relationship between osteoarthritis and fabellar prevalence. In their study, fabellae were present in 35% of knees with primary osteoarthritis and only in 15% of knees in the age-matched control group, which indicated that osteoarthritic knees were prone to present with fabellae^7^. We used a radiological method and showed similar results indicating that OA knees had a higher prevalence of ossicular fabellae than that of those without OA.

Fabella is one part of the knee structure that can degenerate with chondromalacia and osteophyte formation^26^. Clarke AM *et al*. suggested that a “fourth” compartment of the knee is located between the fabella and posterior lateral femoral condyle^32^. This study was the first to show that the degeneration of fabellae was correlated with knee osteoarthritis, which would be helpful for the diagnosis of knee pain, common fibular nerve palsy and especially knee pain following arthroplasty.

TKA has been the most popular and successful surgical treatment for advanced knee osteoarthritis. Worldwide, the number of patients who received TKA is still increasing rapidly^33^. Although most patients are satisfied with the surgical effects, including pain relief, limb alignment correction and increased knee range of motion, some patients complained of knee pain after TKA, which prompted orthopaedists to study the possible reasons, including fabellar disorders, such as fabellar impingement, fracture and arthritic fabella^34,35^. These fabellar disorders can lead to postoperative pain, swelling, and catching.

Fabella could impact both femoral and tibial components of knee prostheses^36^. Jaffe FF *et al*. first reported that an enlarged fabella affected the posterior rim of the tibial component, while the knee was flexed to approximately 90 degrees and caused pain in the posterolateral part of the knee post arthroplasty^14^. Wang JW *et al*. reported that a large fabella caused knee pain after TKA and that the symptoms were diminished after removing the fabella^37^. Fabella fracture after TKA occurred due to chronic accumulated stress, suddenly increased stress on the posterolateral ligamentous complex or the contraction of the gastrocnemius muscle after the correction of valgus malalignment^15,38^. The arthritic fabella could also lead to knee pain due to fabella syndrome after TKA^39^.

Preoperative TKA planning, which includes acquiring a detailed disease history, assessing the symptoms and performing specific tests, and meticulous radiological evaluation, is beneficial for preventing these potential complications. First, if patients complain about posterolateral knee pain preoperatively, further tests concerning fabellar lesions should be conducted. Second, in the lateral aspect of popliteal fosse, the fabella is a hard and mobile mass articulated with a lateral femoral condylar. The knee pain can be irritated with a degenerated fabella through the fabella press test, using one thumb palpate and pressing the fabella and then moving the knee from flexion to extension. A nerve irritative sign, knee pain or numbness of the lateral knee area can also be induced by full knee extension or overextension. Third, meticulous radiological evaluation of the preoperative lateral radiograph of the knee, whether an enlarged (diameter larger than 1 cm) or a severe arthritic fabella exists.

Intraoperatively, after femoral and tibial bone resection, with knee flexion at 90 degrees, fabella can usually be found behind the popliteus muscle tendon, attached with the posterolateral head of the gastrocnemius muscle. The osteophyte formation and sclerosis of the articulated facet could be easily recognized. Excising the fabella and releasing the fabellofibular ligament could help gap balance if the medial gap is larger than the lateral gap. During trail reduction, the careful assessment of fabellar impingement against prostheses by inspection and palpation will help to make a decision on whether to retain or excise the fabella during knee arthroplasty^36,39^.

## Conclusion

In this study, we concluded that fabellar degeneration was correlated with age and knee osteoarthritis. Further studies on the mechanisms of fabellar development, ossification and degeneration, the situation under which the fabella should be removed during TKA, and how the fabella influences the posterolateral structure stability of the knee are encouraged.

## References

[1] Mottershead S (1988). Sesamoid bones and cartilages: An enquiry into their function. Clinical anatomy.

[2] Hauser NH, Hoechel S, Toranelli M, Klaws J, Müllergerbl M (2015). Functional and Structural Details about the Fabella: What the Important Stabilizer Looks Like in the Central European Population. Biomed Research International.

[3] Kawashima T, Takeishi H, Yoshitomi S, Ito M, Sasaki H (2007). Anatomical study of the fabella, fabellar complex and its clinical implications. Surgical & Radiologic Anatomy.

[4] Silva (2010). Morphological Analyisis of the Fabella in Brazilians. International Journal of Morphology.

[5] Zeng SX (2012). Anatomic study of fabella and its surrounding structures in a Chinese population. Surgical and radiologic anatomy: SRA.

[6] Minowa T (2004). Does the fabella contribute to the reinforcement of the posterolateral corner of the knee by inducing the development of associated ligaments?. Journal of Orthopaedic Science.

[7] Pritchett JW (1984). The incidence of fabellae in osteoarthrosis of the knee. Journal of Bone & Joint Surgery-american Volume.

[8] Goldenberg RR, Wild EL (1952). Chondromalacia fabellae. Jbjs.

[9] Oshida M (2012). Two Cases of Fabello-femoral Osteoarthritis That Required Surgical Treatment and the Results of an Anatomical Study of Fabello-femoral Osteoarthritis in Aged Cadavers. *Japanese*. Journal of Rheumatism & Joint Surgery.

[10] Franceschi F (2007). Dislocation of an enlarged fabella as uncommon cause of knee pain: a case report. Knee.

[11] Cherrad T, Louaste J, Bousbaä H, Amhajji L, Khaled R (2015). Fracture of the Fabella: An Uncommon Injury in Knee. Case Reports in Orthopedics,2015, (2015-9-13).

[12] Driessen A (2014). The fabella syndrome - a rare cause of posterolateral knee pain: a review of the literature and two case reports. BMC Musculoskeletal Disorders.

[13] Ando, Y. *et al*. A case report on a very rare variant of popliteal artery entrapment syndrome due to an enlarged fabella associated with severe knee osteoarthritis. *Journal of Orthopaedic Science* (2015). 10.1016/j.jos.2015.06.025 26740435

[14] Jaffe FF, Kuschner S, Klein M (1988). Fabellar impingement: a cause of pain after total knee replacement. A case report. Journal of Bone & Joint Surgery American Volume.

[15] Kwee TC, Heggelman B, Gaasbeek R, Nix M (2016). Fabella Fractures after Total Knee Arthroplasty with Correction of Valgus Malalignment. Case Reports in Orthopedics,2016,(2016-6-1).

[16] Okano E (2016). Fabella Syndrome as an Uncommon Cause of Posterolateral Knee Pain after Total Knee Arthroplasty: A Case Report and Review of the Literature. Case Reports in Orthopedics.

[17] Kellgren JH, Lawrence JS (1957). Radiological Assessment of Osteo-Arthrosis. Annals of the Rheumatic Diseases.

[18] Jin, Z. W. *et al*. A new insight into the fabella at knee: the fetal development and evolution. *Folia morphologica* (2016). 10.5603/FM.a2016.0048 27665955

[19] Mérida‐Velasco JA, Jiménez‐Collado J (1997). Development of the human knee joint. Anatomical Record-advances in Integrative Anatomy & Evolutionary Biology.

[20] Sarin VK, Erickson GM, Giori NJ, Bergman AG, Carter DR (1999). Coincident development of sesamoid bones and clues to their evolution. Anatomical Record.

[21] Kaplan EB (1961). The fabellofibular and short lateral ligaments of the knee joint. Journal of Bone & Joint Surgery-american Volume.

[22] Raheem O, Philpott J, Ryan W, O’Brien M (2007). Anatomical variations in the anatomy of the posterolateral corner of the knee. Knee Surgery Sports Traumatology Arthroscopy.

[23] Yu JS (1996). Posterolateral aspect of the knee: improved MR imaging with a coronal oblique technique. Radiology.

[24] Terry GC, Laprade RF (1996). The posterolateral aspect of the knee. Anatomy and surgical approach. American Journal of Sports Medicine.

[25] Piyawinijwong, S., Sirisathira, N. & Sricharoenvej, S. The fabella, fabellofibular and short lateral ligaments: an anatomical study in Thais cadavers. *Siriraj Medical Journal* (2016).

[26] Ehara S (2014). Potentially symptomatic fabella: MR imaging review. Japanese Journal of Radiology.

[27] Chew CP, Lee KH, Koh JS, Howe TS (2014). Incidence and radiological characteristics of fabellae in an Asian population. Singapore Med J.

[28] Iida H, Arisawa H, Yufu J (1976). A case report of peroneal nerve palsy compressed by the fabella. Orthop Surg.

[29] Phukubye P, Oyedele O (2011). The incidence and structure of the fabella in a South African cadaver sample. Clinical anatomy.

[30] Tabira Y (2013). Influence of a fabella in the gastrocnemius muscle on the common fibular nerve in Japanese subjects. Clinical anatomy.

[31] Egerci OF (2017). Prevalence and distribution of the fabella: a radiographic study in Turkish subjects. Folia morphologica.

[32] Clarke AM, Matthews JG (1991). Osteoarthritis of the fabella: a fourth knee compartment?. Journal of the Royal College of Surgeons of Edinburgh.

[33] Mccalden, R. W. *et al*. Clinical Results and Survivorship of the GENESIS II Total Knee Arthroplasty at a Minimum of 15 Years. *Journal of Arthroplasty* (2017). 10.1016/j.arth.2017.02.006 28285899

[34] Ali A (2014). Dissatisfied patients after total knee arthroplasty. Acta Orthopaedica.

[35] Seah RB (2016). Unexplained pain post total knee arthroplasty with an Oxford knee score ≥20 at 6 months predicts good 2 year outcome. Journal of Arthroplasty.

[36] Larson JE, Becker DA (1993). Fabellar impingement in total knee arthroplasty. A case report. Journal of Arthroplasty.

[37] Wang JW (1995). Fabellar impingement after total knee replacement–a case report. Changgeng Yi Xue Za Zhi.

[38] Theodorou SJ, Theodorou DJ, Resnick D (2005). Painful stress fractures of the fabella in patients with total knee arthroplasty. Ajr American Journal of Roentgenology.

[39] Laird L (1991). Fabellar joint causing pain after total knee replacement. Journal of Bone & Joint Surgery British Volume.

